# Labor force participation and secondary education of gender inequality index (GII) associated with healthy life expectancy (HLE) at birth

**DOI:** 10.1186/s12939-014-0106-2

**Published:** 2014-11-18

**Authors:** Jong In Kim, Gukbin Kim

**Affiliations:** Division of Health and Welfare, Wonkwang University, Iksan-si, Republic of Korea; Management with International Business (BSc), Royal Holloway, University of London, London, UK

**Keywords:** Gender Inequality Index, Healthy life expectancy, Secondary education, Labor force participation

## Abstract

**Background:**

What is the factor that affects healthy life expectancy? Healthy life expectancy (HLE) at birth may be influenced by components of the gender inequality index (GII). Notably, this claim is not tested on the between components of the GII, such as population at least secondary education (PLSE) with ages 25 and older, labor force participation rate (LFPR) with ages 15 and older, and the HLE in the world’s countries. Thus, this study estimates the associations between the PLSE, LFPR of components of the GII and the HLE.

**Methods:**

The data for the analysis of HLE in 148 countries were obtained from the World Health Organization. Information regarding the GII indicators for this study was obtained from the United Nations database. Associations between these factors and HLE were assessed using Pearson correlation coefficients and regression models.

**Results:**

Although significant negative correlations were found between HLE and the LFPR, positive correlations were found between HLE and PLSE. Finally, the HLE predictors were used to form a model of the components of the GII, with higher PLSE as secondary education and lower LFPR as labor force (R^2^ = 0.552, P <0.001).

**Conclusions:**

Gender inequality of the attainment secondary education and labor force participation seems to have an important latent effect on healthy life expectancy at birth. Therefore, in populations with high HLE, the gender inequalities in HLE are smaller because of a combination of a larger secondary education advantage and a smaller labor force disadvantage in male-females.

## Background

The healthy life expectancy (HLE) summarizes mortality and non-fatal outcomes in a single measure of average population health. The HLE increases more slowly than life expectancy [1]. The HLE is a related statistic that estimates the equivalent years that a person can expect to live in full health based on the current mortality rates and the prevalence distribution of health states in the population [2].

The HLE index was first used as a measure by the WHO in 2000 [3], combining information on mortality and morbidity [4]. However, the HLE - related quality of life, sometimes called health-adjusted life expectancy, is a health expectancy indicator that expands measures of life expectancy to represent the average health in a population, in the terms of equivalent years of full health, taking into account the distribution of health states [5]. Thus, the HLE is defined as an estimate of the average number of years that a person can expect to live in full health, by taking into account years lived in less than full health due to disease and/or injury [6]. The resulting studies of the HLE have been conducted in some countries [1,3-5,7-10]. However, the studies for secondary education and labor force participation of components of the gender inequality index (GII) have a few examined on the effects associated with HLE [7-10]. The retrospective analysis of those factors that contribute to the HLE may help identify factors associated with the GII. Thus, the HLE has been used to compare full health between countries [1]. These comparisons can inform policy questions regarding equality of human rights that depend on components of the GII. We consider how the HLE correlates with components of GII.

In the course of the twentieth century, the overall mortality reduction was more beneficial for women and resulted in a substantial widening of the male and female longevity gap [7]. Until today, life expectancy of women exceeds that of men, although the size of the gender gap varies between populations [8]. However, the study showed that due to the combination of the higher prevalence of disability and lower mortality women spent substantially more years with disability than men [7,9]. Health expectancies, predominantly disability-free life expectancy, are available for many countries worldwide [10].

Thus, gender inequalities in HLE, can be split into two components of GII: (1) the inequality in secondary education and (2) in labor force participation. The current study is to better understand the secondary education and labor force participation of GII within the 148 countries by examining the contribution of women’s HLE advantage or disadvantage. We expect that in populations with high HLE, the gender inequalities in HLE are smaller because of a combination of a larger secondary education advantage and a smaller labor force disadvantage in females.

To reduce gender inequalities, insight is needed into the underlying causes of the inequalities, as these may point at possible interventions, either at the societal level, to reduce them [7]. Though several biological hypotheses have been proposed, the dynamics of the gender differences in mortality suggest that its determinants cannot be purely biological, but are also dependent on modifiable psychosocial and lifestyle factors [10].

Although knowledge regarding the determinants of health is limited, full health is a multifactorial quantitative trait that is influenced by biological, environmental, and psychosocial factors [11]. Among all these elements, the components of the GII, as modifiable risk factors, have not been studied in relation to the HLE of full health. Briefly, although studies have shown that educational factors, such as educational inequalities [12-17], can predict morbidity in incidences of disease, but it is currently uncertain whether these associations are applicable to the HLE and percentage of population at least secondary education (PLSE) with ages 25 and older as empowerment of components of GII. Furthermore, the association between educational attainment status, at least secondary education in ages 25 and older, and the HLE of full health and empowerment has not been studied [12-19].

Meanwhile, although studies have shown that labour force factors, such as labor force participation [20-25], can predict morbidity, this study was designed to test whether these associations are applicable to the HLE and percentage of population labor force participation rate (LFPR) with ages 15 and older as labour market of GII. Furthermore, although there have been studies investigating the effect of child labor on the growth of children [26-29], the association between labor force participation with ages 15 and older and the HLE has not been studied. We are uncertain whether these labor associations are applicable to the HLE.

The HLE may be influenced by components of the GII. Notably, this claim is not tested on the between components of the GII such as PLSE, LFPR and HLE in the world’s countries. Thus, this study estimates the associations between the PLSE, LFPR and HLE in 148 countries.

## Methods

### The framework of HLE and components of GII

The HLE index expands measures of life expectancy to represent the average health in a population in terms of equivalent years of full health [5]. However, the GII measures gender inequalities in three important aspects of human development [30]. In other words, GII measures reflecting inequality in achievements between women and men in three dimensions and five indicators: reproductive health (maternal mortality and adolescent fertility), empowerment (parliamentary representation and secondary education attainment) and the labor market (labor force participation) [30,31]. Thus, The GII reflects women’s disadvantage in three dimensions for as many countries as data of reasonable quality allow. It ranges from 0, which indicates that women and men fare equally, to 1, which indicates that women fare as poorly as possible in all measured dimensions. The reproductive health dimension is measured by two indicators: maternal mortality ratio (MMR) and adolescent fertility rates (AFR). However, in this study excluded indicator of MMR and AFR because that the HLE is a measure of mortality and morbidity with another measure of mortality and morbidity; MMR is the same as correlating mortality with mortality and is not scientifically sound. The empowerment dimension is also measured by two indicators: the share of seats in national parliament (SNP) held by female and by educational attainment of secondary level and above percentage of population at least secondary education (PLSE) with ages 25 and older by gender. However, in this study is excluded indicator of SNP since the number of countries covered varies with suspensions or dissolutions of parliaments, there can be difficulties in obtaining information on by-election results and replacements due to death or resignation, and the use national parliamentary representation excludes participation at the local government level and elsewhere in community and public life [30]. The labor dimension is measured by labor force participation rate (LFPR) with ages 15 and older by gender. The GII is designed to reveal the extent to which national achievements in these aspects of human development are eroded by gender inequality, and to provide empirical foundations for policy analysis and advocacy efforts [30,31].

**Figure 1:**
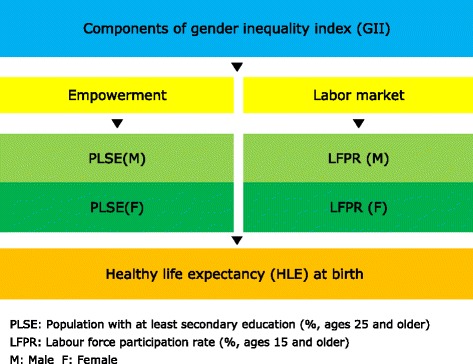
**Description of theoretical framework.**

Therefore, on the assumption that the HLE may be influenced by components of the GII, the framework of this study depicts relationships between the HLE, as life expectancy to full health, and components of the GII reflecting the two dimensions: the PLSE as empowerment measures, and the LFPR as a labor measure (Figure 1).

### Estimation of HLE

The equivalent lost healthy year fractions required for the HLE calculation are estimated as the all-cause years lost due to disability (YLD) rate per capita, adjusted for independent comorbidity, by age, sex and country. Sullivan’s method uses the equivalent lost healthy year fraction at each age in the current population to divide the hypothetical years of life lived by a period life table cohort at different ages into years of equivalent full health and equivalent lost healthy years [6]. However, the first challenge is lack of reliable data on mortality and morbidity, especially from low-income countries. Other issues include lack of comparability of self-reported data from health interviews and the measurement of health-state preferences for such self-reporting [6]. Thus, concerning the year, the HLE in this study reflects the [Male and Female (MF)], [Male (M)], and [Female (F)] for the year 2007 as a retrospective study.

### Hypothesis and setting model

In order to examine the association between HLE disparities and the components of the GII, we need to develop a study model with each variable. This model was used to estimate HLE in terms of the components of the GII. The models depict the framework proposed herein of the components of the GII, according to the variables selected. The three models [HLE (MF), HLE (M) and HLE (F)] yielded the following results. The HLE predictors were used to form a combination model, from model [HLE (MF)] to model [HLE (F)] of the full health factors with PLSE as empowerment and LFPR as labor market factors. These variables are reflective of the components of the GII. Thus, these indirectly packing factors [PLSE (F) and LFPR (F)] to full health may differ for healthy life expectancy. Therefore, from this model, we derived a hypothesis stating that increases in the PLSE (F), and decreases in the LFPR (F) will lead to a corresponding increase in the HLE. Thus, associations between these factors and HLE of from this model were assessed using Pearson correlation coefficients and regression models.

### Data collection for the HLE and GII

This study utilized the demographic databases of 148 countries for calculations. The data for the analysis of the HLE were obtained from the healthy life expectancy at birth conducted by World Health Organization (WHO) [32]. The countries and overseas island dependencies were selected according to the classification system applied by the United Nations. The 148 countries were selected in the more and less developed regions. The components of the GII for this study were obtained from a dataset in the United Nations database [33]. The following factors were used: (1) the PLSE (percentage, ages 25 and older) of GII, 2010; (2) the LFPR (percentage, ages 15 and older) of GII, 2011.

### The HLE, PLSE and LFPR disparities

Table 1 presents the descriptive statistics for this range of HLE along with the components of the GII such as PLSE and LFPR indicators. Generally, the HLE (MF) - related quality of life in 148 countries ranged from 35, to 76, with a mean of 60.58. The HLE (MF) disparity between the countries was 41 years. The HLE (M) - related quality of life ranged from 34 to 73, with a mean of 59.11. However, the HLE (F) ranged from 36 to 78, with a mean of 62.09. The HLE (F) disparity between the countries was 42 years. Meanwhile, the PLSE (F) ranged from 0.9 to 100, with a mean of 53.16. The PLSE (F) disparity between the countries was 99.1%. Lastly, the LFPR (F) ranged from 13.1 to 88.2, with a mean of 52.68. The LFPR (F) disparity between the countries was 75.1%. Therefore, the descriptive statistics for this range of HLE indicators are presented the higher HLE (Female) than HLE (Male) in 148 countries. The HLE (Male) and HLE (Female) disparity between the countries was 2.98 years.

**Table 1:** **Descriptive statistics of variable**

**Variable**	**N**	**Mean**	**StDev** ^**a**^	**Minimum**	**Maximum**
HLE (MF)	148	60.58	9.98	35	76
HLE (M)	148	59.11	9.623	34	73
HLE (F)	148	62.09	10.49	36	78
PLSE (M)	148	58.62	27.8	3.2	100
PLSE (F)	148	53.16	30.67	0.9	100
LFPR (M)	148	74.78	8.052	45.1	95.2
LFPR (F)	148	52.68	16.24	13.1	88.2

^a^Standard deviation.

HLE: Healthy life expectancy at birth.

PLSE: Population with at least secondary education (% ages 25 and older).

LFPR: Labour force participation rate (% ages 15 and older).

MF: Male and Female, M: Male, F: Female.

## Results

### The prediction variables of HLE

**Figure 2:**
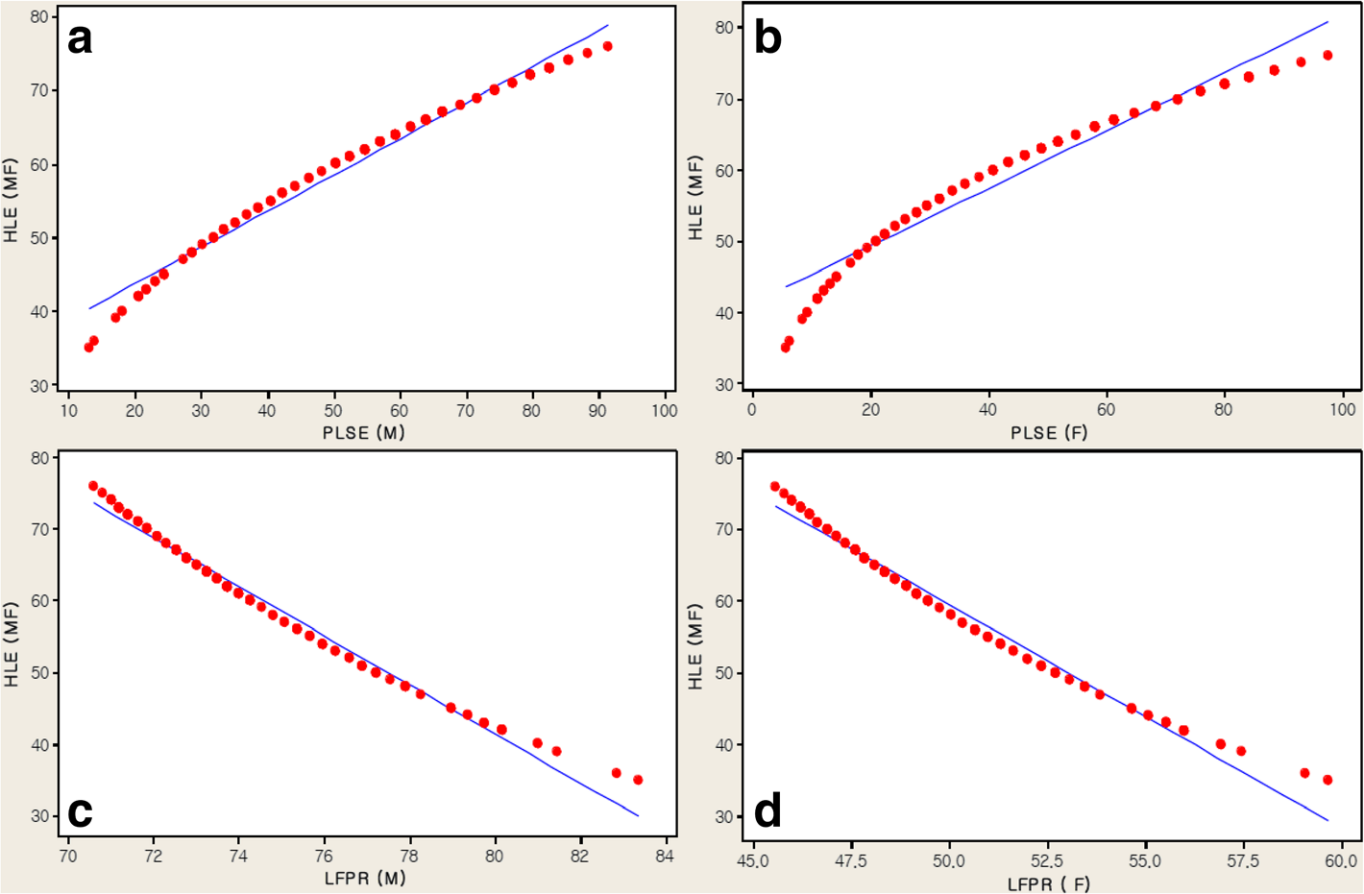
**HLE (MF) associated with PLSE and LFPR. a**. HLE (MF) associated with PLSE (M). **b**. HLE (MF) associated with PLSE (F). **c**. HLE (MF) associated with LFPR (M). **d**. HLE (MF) associated with LFPR (F).

**Figure 3:**
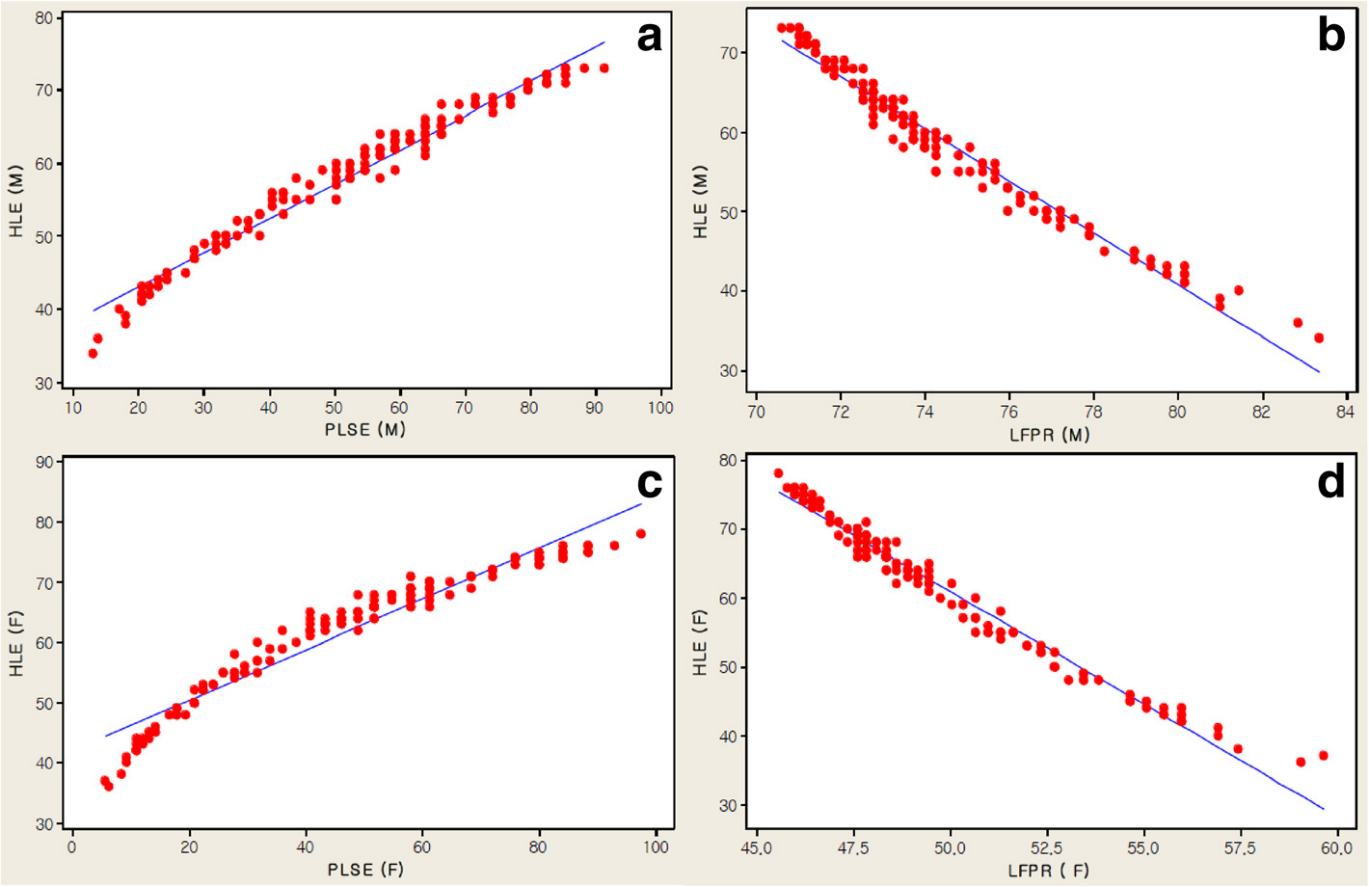
**HLE (M and F) associated with PLSE and LFPR. a**. HLE (M) associated with PLSE (M). **b**. HLE (M) associated with LFPR (M). **c**. HLE (F) associated with PLSE (F). **d**. HLE (F) associated with LFPR (F).

Table 2, Figure 2a-d and Figure 3a-d and Table 3 present the analysis of the PLSE and the LFPR factors related to the HLE in 148 countries.

**Table 2:** **Correlations Coefficient for HLE (MF), HLE (M) and HLE (F)**

**Variable**	**HLE (MF)**		**HLE (M)**		**HLE (F)**	
	**Coefficient**	**P-value**	**Coefficient**	**P-value**	**Coefficient**	**P-value**
PLSE (M)	0.677	0.001	0.652	0.001	0.687	0.001
PLSE (F)	0.699	0.001	0.672	0.001	0.710	0.001
LFPR (M)	-0.368	0.001	-0.326	0.001	-0.397	0.001
LFPR (F)	-0.270	0.001	-0.271	0.001	-0.276	0.001

HLE: Healthy life expectancy at birth.

PLSE: Population with at least secondary education (% ages 25 and older).

LFPR: Labour force participation rate (% ages 15 and older).

MF: Male and Female, M: Male, F: Female.

**Table 3:** **Prediction variables for HLE (MF), HLE (M) and HLE (F)**

**Predictions variable**		**Coefficient**	**T-value**	**P-value**	**R** ^**2**^
HLE (MF)	PLSE (F)	0.221	11.94	0.001	0.532
	LFPR (F)	-0.131	-3.71	0.001	
HLE (M)	PLSE (F)	0.205	11.07	0.001	0.498
	LFPR (F)	-0.128	-3.65	0.001	
HLE (F)	PLSE (F)	0.237	12.38	0.001	0.552
	LFPR (F)	-0.141	-3.88	0.001	

HLE: Healthy life expectancy at birth.

PLSE: Population with at least secondary education (% ages 25 and older).

LFPR: Labour force participation rate (% ages 15 and older).

MF: Male and Female, M: Male, F: Female.

In the interactions between education and labor force participation, the PLSE (M) was correlated with the LFPR (M) as components of the GII (r = -0.557, P =0.001, N =148). Although significant negative correlations were found between the PLSE (M) and the LFPR (M), non-correlations were found between the PLSE (F) and LFPR (F) in 148 countries.

The HLE, including male and female, for all 148 countries was correlated with the PLSE and the LFPR as components of the GII. Although significant negative correlations were found between the HLE and the labor force participation of LFPR (M) and LFPR (F), positive correlations were found between the HLE and educational attainment at least secondary level of PLSE (F) and PLSE (M) in 148 countries (Table 2). Here we consider comparing relative ratios. These measures are analyzed on the log scale. A log scale was used for all explanatory variables (see Figures 2a-d and 3a-d).

In order to investigate the direct relationships between the components of the GII such as the PLSE and the LFPR indicators and the HLE for all 148 countries, we conducted a multiple regression analysis. The regression analysis of the components of the GII found the strongest predictors among the three regression models (Table 3). The HLE (MF) predictors were used to form a model of the components of the GII, with lower LFPR (F) and higher PLSE (F) in 148 countries (R^2^ = 0.532, P <0.001). Finally, the HLE (F) predictors were used to form a model of the components of the GII, with lower LFPR (F) and higher PLSE (F) (R^2^ = 0.552, P <0.001).

## Discussion

Gender inequality remains a major barrier to human development. Females have made major strides since 1990, but they have not yet gained gender equity [30]. The disadvantages facing women and girls are a major source of inequality. All too often, females are discriminated against in education and labour market with negative repercussions for development of their capabilities and their freedom of choice [30]. Thus, we considered the associations between the HLE and the components of the GII such as the PLSE and the LFPR indicators to examine whether the lower HLE were disproportionately susceptible to full health.

*The empowerment factors* such as PLSE, which can contribute to healthy living, as indicators of women’s HLE advantage. Increases in the PLSE, as components of the GII, led to an increase in HLE values, suggesting that they are significant contributory factors to the HLE in 148 countries. In the current study, the PLSE scores were the lowest in the less developed regions of Africa, whereas those scores were the highest in more developed regions, among any of the countries studied. This study have shown that educational attainment level, such as PLSE, as empowerment of components of GII, could predict the HLE, like that educational inequalities [12-17]. As such, the PLSE is likely to be a major contributing factor to higher HLE in 148 countries. Therefore, the PLSE (Female) that indirectly reflects the women’s empowerment factors necessary for healthy living was to be significant factors of HLE (Female). This means that if the HLE factors associated with women’s empowerment improve by increasing female enrollment in secondary education, uplifting their social and political status with a greater share of female seats in the national parliament, then their healthy living will also improve. Thus, gender equity is also a crucial determinant of health inequalities at the national level, and is important for the surveillance of women’s and men’s health, as well as for future health policy initiatives [34]. This is particularly important because of the associations that exist between gender equality and empowerment among women [35]. Furthermore, women’s right to health has been reiterated many times. However, there are social and cultural barriers in developing countries that hinder their empowerment [36,37]. On the other hand, the high correlations found in the current study between the HLE, and PLSE as components of the GII have bearing on government policies, since these variables are reflective of the government’s investment in sociocultural education infrastructure for healthy living. Change needs to happen on the political and cultural level if there is to be gender equality in more developed countries. Therefore, the HLE (Female) level seems to have an important latent effect on women’s empowerment, improving in relation to female enrollment in secondary education and to their social status.

*The labor market factor* is an indicator of labor force participation, which can contribute to healthy living. Decreases in LFPR, as a component of the GII, led to an increase in the HLE values, suggesting that LFPR is a significant contributory factor to the HLE. In the current study, the LFPR in Eastern Europe were the lowest among all the 148 countries, whereas those of East Africa were the highest. As such, the LFPR is likely to be a major contributing factor to a high HLE. The decreases in LFPR (Female) led to an increase in the HLE, suggesting that LFPR (Female) is a significant factor of HLE (Female). The LFPR (F) as a component of the GII, which reflects the labor force participation level for those aged 15 and older, was to be significant factor of HLE in less developed regions. In the current study has shown that labour force level, such as LFPR labour market of components of GII, could predict the HLE, like that labor force participation [20-25]. For example, the labour force participation has a significant negative effect on younger males’ health [38]. Furthermore, the higher the rate of labor force participation for those aged 15 and older, the less value of the HLE (Male-Female) will be, with the result that poor health increased the risk of disability pension, but was not related to early retirement [39]. A lack of physical activity could be a determinant of disability pension and unemployment [39,40]. Therefore, in this study, the lower LFPR (Male-Female) is an important independent contributor to higher HLE (Male-Female) of healthy life.

In the HLE calculation of WHO, the limitation of this study is lack of comparability of self-reported data from health interviews and the measurement of health-state preferences for such self-reporting [6]. In some countries, estimates of HLE are subject to the uncertainty, especially for countries with weak statistical and health information systems where the quality of underlying empirical data is limited [32]. The GII of this study is excluded indicator of share of seats in national parliament (SNP) in empowerment and reproductive health (MMR and AFR). However, it is important to clarify that the direct relationships between the components of the GII such as the PLSE and the LFPR indicators and the HLE.

Hence, the hypothesis that if the countries for the proposed three models [HLE (Male-Female), HLE (Male) and HLE (Female)] were to have lower LFPR (Female) and higher PLSE (Female), the associations between components of the GII and HLE could predict a certain impact on the increase of the HLE. In other words, HLE is a state of gender equality in labor force participation and secondary education. Furthermore, the HLE is based on the healthy longevity or survival probability of becoming a healthy centenarian [41,42].

## Conclusion

Gender inequality of the attainment secondary education and labor force participation seems to have an important latent effect on healthy life expectancy at birth. Therefore, in populations with high HLE, the gender inequalities in HLE are smaller because of a combination of a larger secondary education advantage and a smaller labor force disadvantage in females.
